# Adolescent with severe granulomatosis with polyangiitis: a case report

**DOI:** 10.11604/pamj.2021.38.285.26893

**Published:** 2021-03-18

**Authors:** Hajar Arfaoui, Hamza Elkihal, Hasna Jabri, Wiam Elkhattabi, Hicham Afif

**Affiliations:** 1 Department of Respiratory Diseases, Hospital 20 Août 1953, University of Hassan II, University Hospital Center Ibn Rochd, Casablanca, Morocco

**Keywords:** Granulomatosis with polyangiitis, Wegener’s granulomatosis, pediatric GPA, antineutrophil cytoplasmic antibody -associated vasculitis, case report

## Abstract

Granulomatosis with polyangiitis (GPA) is a rare vasculitis among adolescents. Its pulmonary manifestations may mimic tuberculosis. We report the case of a 16-years-old female patient with multiple excavated lung nodules revealed by a chronic cough, hemoptysis, epistaxis and weight loss. The diagnosis of GPA was achieved due to systemic pulmonary, ENT and renal involvement, the positivity of anti-neutrophil cytoplasmic antibody directed against proteinase 3 (C-ANCA) and bronchial and nasal biopsies showing granulomatous inflammation with a dense perivascular infiltrate destroying the vessel wall. Bolus of glucocorticoids and immunosuppressants reversed her symptoms. Although GPA is a rare disease in teenagers, it should be considered as one of the differential diagnosis in adolescents presenting with excavated pulmonary nodules.

## Introduction

Granulomatosis with polyangiitis (GPA) is a systemic vasculitis, associated with the presence of anti-neutrophil cytoplasmic antibody (ANCA) [1]. It can affect any organ system with potential life-threatening morbidities. It has a low incidence in the pediatric population [2]. We present a rare case of an adolescent with GPA involving pulmonary, renal and nasal manifestations.

## Patient and observation

**Figure 1:**
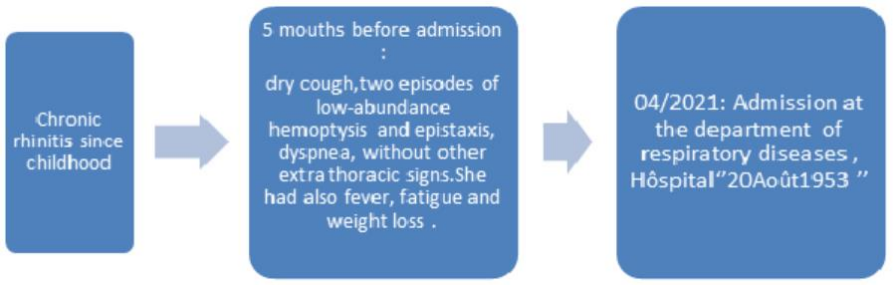
timeline of this case

A 16 years old female, with a history of chronic rhinitis, had presented, for the past 5 months, a dry cough with two episodes of low-abundance hemoptysis, a progressive worsening dyspnea and epistaxis. She had fever, fatigue and weight loss without other extra pulmonary manifestations (Figure 1). On examination, her pulse was 124 beats/min, blood pressure was 120/60mmHg, the temperature was 37°C and the oxygen saturation was 91% while she was breathing ambient air. The urine dipstick found hematuria (+) without proteinuria. Pulmonary examination revealed bilateral sibilant rales without crackling. Other systems examination revealed no significant abnormalities.

**Figure 2:**
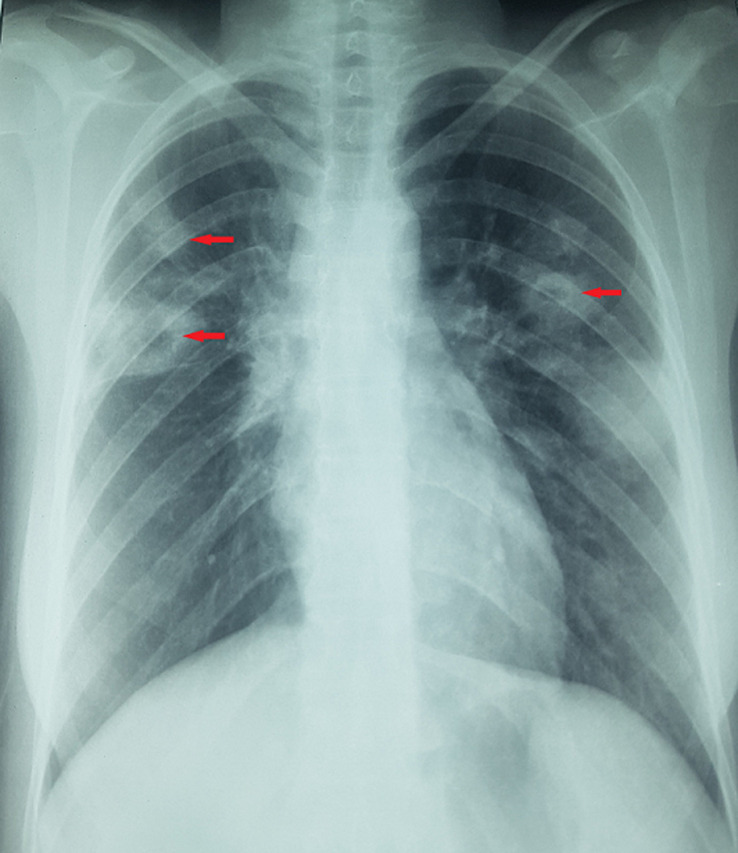
chest X-ray showing bilateral excavated opacities in the upper lungs

**Figure 3:**
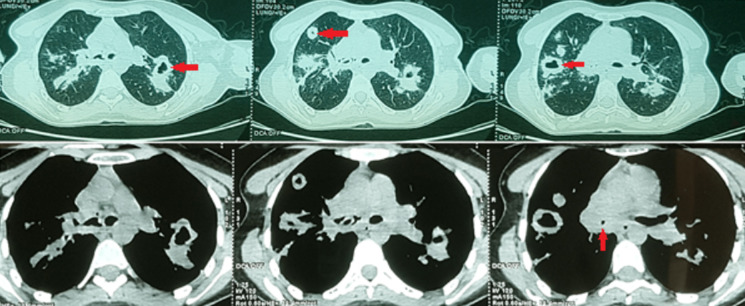
CT scan with multiple bilateral excavated nodules, reduction in the right main bronchus caliber with mediastinal lymphadenopathy

**Figure 4:**
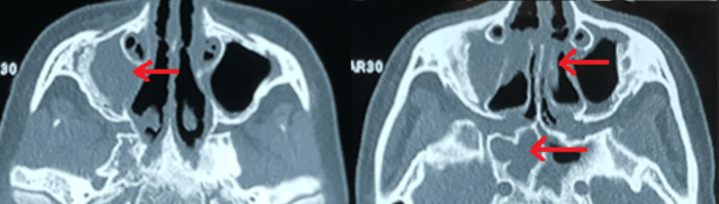
CT scan of the sinus showing filled maxillary and sphenoid sinuses with wall thickening, a polypoid mass of the right middle meatus and nasal septum erosion

**Figure 5:**
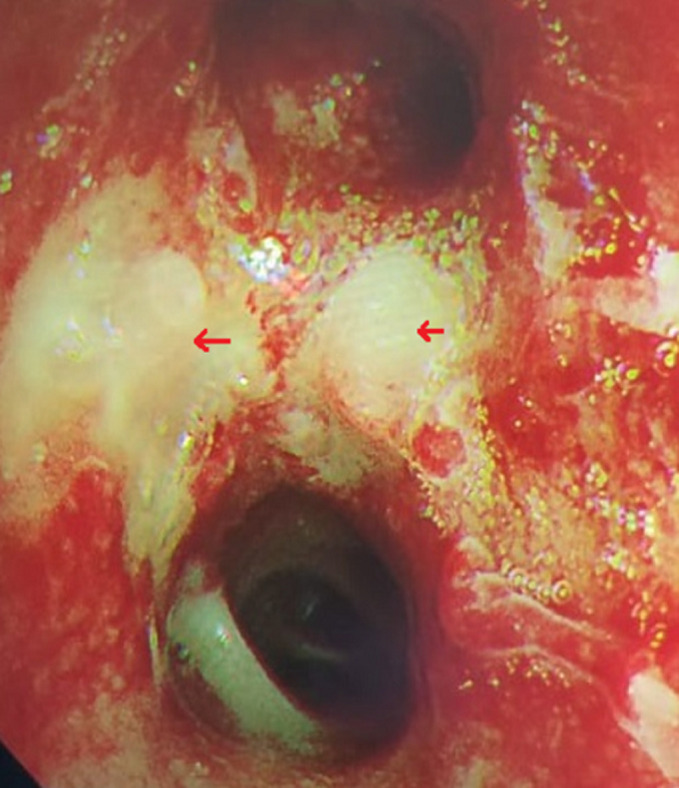
bronchoscopy showing inflammation and infiltration of the bronchial tree, covered with whitish granulations

Chest X-ray showed bilateral excavated opacities in the upper lungs (Figure 2). The chest computed tomography (CT) found multiple bilateral excavated nodules with the reduction of the right main bronchus caliber and mediastinal lymphadenopathy (Figure 3). The CT-scan of the sinus showed filled maxillary and sphenoid sinuses with wall thickening, a polypoid mass of the right middle meatus and nasal septal erosion (Figure 4). The blood tests showed microcytic hypochromic anemia 11.1 g/dl, elevated CRP 109 mg/l, erythrocyte sedimentation rate (ESR) 68mm, normal urine proteinuria excretion 7mg/24 hours, urea 0,32g/l,serum creatinine 7.5mg/l and glomerular filtration rate GFR at 133 ml/min/1.73m^2^. Cyto-bacteriological examination of urine was sterile with microscopic hematuria 17x104 red blood cell (RBC)/ml. The tests for viral hepatitis B, C and HIV were negative. The electrocardiography (ECG) and cardiac ultrasound were normal. The bronchoscopy revealed an infiltration of the entire bronchial tree which was covered by whitish granulations, with narrowing of the orifice of the intermediate trunk, as well as the bronchi of the upper right lobe (Figure 5).

The research of acid-fast bacilli (AFB) and other bacteria like actinomyces and fungus in bronchial aspiration culture were negative. The biopsies of the bronchus and the granulations showed tuberculoid granuloma inflammation without caseous necrosis. The rhinocavoscopy showed a significant inflammation of the turbinates with the presence of whitish granulations and synechiae. The biopsy of the middle turbinate and the cavum revealed a granulomatous inflammation without caseous necrosis and a dense peri-vascular infiltrate, destroying the vessel wall, composed of neutrophils with fibrinoid deposits. The anti-proteinase 3 anti-neutrophil cytoplasmic antibodies (ANCA-Pr3) was positive. The anti-myeloperoxidase anti-neutrophil cytoplasmic antibodies (ANCA-MPO) was negative. Granulomatosis with polyangiitis also called Wegener´s granulomatosis was diagnosed on the basis of pulmonary, nasal and renal involvement, the positivity of anti-neutrophil cytoplasmic antibody directed against proteinase 3 (ANCA-Pr3 or C-ANCA), the bronchial and nasal biopsies that confirm the granulomatosis and after eliminating infectious diseases such as tuberculosis.

Regarding the diffuse bilateral pulmonary involvement with significant inflammation and narrowing of the bronchial orifices, as well as the Ear-Nose-Throat (ENT) involvement, and the hematuria considered as incipient renal lesion, the bolus of methyl prednisolone were started (15mg/kg/day) for 3 successive days followed by corticosteroid therapy prednisone (1mg/kg/day). Adjuvant treatment including calcium and vitamin D was prescribed. Due to the risk of infertility after exposure to cyclophosphamide (CYC) and the high price of rituximab (RTX), we opted for azathioprine bolus. The symptoms reversed after the second bolus of corticosteroids by decreasing of dyspnea and regaining appetite. Currently, the patient is still in remission phase.

## Discussion

The Granulomatosis with polyangiitis (GPA), known as Wegener´s granulomatosis [1], is recognized as primary systemic vasculitis of small blood vessels [2]. Usually it occurs between 45 and 60 years of age, with a peak in the sixth decade but in a small proportion of cases (3.3 - 7%), it may affect also children and adolescents [3]. Incidence of juvenile onset GPA is not well known but estimates range from 0.02 to 0.64 per 100,000 persons per year [4, 5]. Pediatric GPA is usually diagnosed in adolescence, with a median age at onset of 11.6 years and a median age at diagnosis of 14 years. It presents a female predominance with a male-to-female ratio of 1: 2.1 [6, 7].

The pathogenesis of GPA is not entirely clear. Genetic susceptibility factors, environmental agents, as well as abnormalities in innate and adaptive immune responses appear to contribute to the development of GPA [8]. It is recognized as a primary systemic vasculitis of small blood vessels, characterized by the presence of inflammation affecting the blood vessel wall, resulting in tissue ischemia and necrosis. Patients with GPA are typically positive for PR3-ANCAs (proteinase 3 also called as myeloblastin) [9]. GPA is generally characterized by ear-nose-throat (ENT) involvement followed by constitutional symptoms, renal, lower respiratory tract manifestations. In some cases, necrosis and granulomatous lesions cause destruction of the turbinates and nasal septum perforation. The most frequent lung lesions are nodules tending to excavation, single or multiple, sometimes confluent like in our case. Diffuse alveolar hemorrhage can occur and it is a life-threatening condition [6]. Constitutional symptoms like fever, fatigue, anorexia and weight loss may precede the systemic organ manifestations and are mostly non-specific findings [10]. Renal involvement is noted in up to 80% of patients with GPA and includes hypertension, edema, proteinuria, and hematuria [11]. Other organs lesions can be affected like skin (purpura, livedo reticularis), musculoskeletal symptoms (arthralgia, myalgia) and gastrointestinal (chronic nausea, diarrhea and non-specific abdominal pain) [12].

GPA criteria were defined by the EULAR/PReS/PRINTO joint committee. Three of the following six criteria were required to classify pediatric vasculitis as GPA: histopathology (granulomatous inflammation within the arterial wall or in the perivascular or extravascular area), upper airway involvement (chronic purulent or bloody nasal discharge or recurrent epistaxis/crusts/granulomata, nasal septum perforation or saddle nose deformity, chronic or recurrent sinus inflammation), laryngotracheo-bronchial stenoses (subglottic, tracheal or bronchial stenosis), pulmonary involvement (chest X-ray or CT scan showing the presence of nodules, cavities or fixed infiltrates), ANCA positivity by immunofluorescence or ELISA (P-ANCA/MPO-ANCA or C-ANCA/PR3- ANCA) and renal involvement (proteinuria > 0.3 g/24 h or > 30 mmol/mg of urine albumin/creatinine ratio on a spot morning sample, hematuria or red blood cell casts in the urinary sediment or ≥2+ on dipstick, or necrotizing pauci-immune glomerulonephritis) [13, 14]. Our patient met all the criteria. The development of a validated scoring tool to measure disease activity, damage and outcomes is crucial for pediatric vasculitis. Modification of the vasculitis damage index (PVDI) contains 72 items in 10 systems [15]. Once validated, PVDI should serve as an important step toward better disease assessment in clinical trials in children with systemic vasculitis. No specific pediatric management guidelines are available to guide the therapeutic approach in pediatric patients with GPA. The Canadian vasculitis research network (CanVasc) recommends that children with newly diagnosed GPA should be treated as per adult recommendations for induction of remission and then maintenance [16, 17].

EULAR/ERA-EDTA (European League Against Rheumatism/ European Renal Association-European Dialysis and Transplant Association) and CanVasc recommend treatment with a combination of glucocorticoids and either cyclophosphamide (CYC) or rituximab (RTX) [16, 17]. In life-threatening disease or those with major organ involvement, pulsed IV methylprednisolone 0.5-1 g/day for 3 consecutive days is recommended. In children (< 15 years old), the initial dose of oral prednisone used is 1-2 mg/kg/day with a maximum of 60 mg/day. Daily calcium (500-1000mg) and vitamin D (1000 IU) supplementation is recommended [17]. RTX is preferred as a first line remission induction therapy for patients in whom CYC is contraindicated or presents a risk of infertility. CYC can be administered either orally or as pulse intravenous dose (3-6 months) but the latter is preferred as it is associated with less cumulative dose and reduced risk of bladder-related complications. However, daily oral low-dose CYC is associated with a slightly lower rate of relapse on long-term follow up [18]. For remission maintenance therapy, the CanVasc as well as EULAR/ERA-EDTA recommends treatment with a combination of low-dose glucocorticoids and either azathioprine (AZA), rituximab, methotrexate or mycophenolate mofetil. This therapy for GPA must be continued for at least 24 months following induction of sustained remission. For relapsing disease, both the CanVasc and EULAR/ERA-EDTA guidelines recommend switching from RTX to CYC and vice versa. For patients who continue to have persistent active disease, intravenous immunoglobulin may be used as an adjunctive therapy [16, 17]. The plasmapheresis is recommended to be used for rapidly progressive glomerulonephritis in the setting of new or relapsing disease or for the treatment of severe diffuse alveolar hemorrhage [16, 19]. Mortality rates are low in pediatric GPA and usually do not exceed 5-10% [7, 20].

## Conclusion

Pediatric GPA is a rare systemic vasculitis with life threatening and severe complications. Our case shows the importance of considering the GPA as one of the differential diagnosis amid adolescent presenting with excavated pulmonary nodules. The treatment is extrapolated from the adult studies and long term surveillance is necessary.
